# Stage-specific disruption of erythropoiesis leads to anemia in newly diagnosed multiple myeloma patients

**DOI:** 10.3389/fcell.2026.1718025

**Published:** 2026-06-10

**Authors:** Shihui Wang, Wenna Fu, Changyu Chen, Haoran Kong, Saishuo Liu, Jiejie Guo, Dan Li, Rongjun Ma, Donghao Liu, Hongdan Chen, Xiaoli Qu, Mengke Li, Chao An, Huizhi Zhao, Zunmin Zhu

**Affiliations:** 1 Institute of Hematology, Henan Provincial People’s Hospital, People’s Hospital of Zhengzhou University, Zhengzhou, China; 2 School of Life Sciences, Zhengzhou University, Zhengzhou, China; 3 Department of Orthopedics, Henan Provincial People’s Hospital, People’s Hospital of Zhengzhou University, Zhengzhou, China; 4 Department of Magnetic Resonance Imaging, The First Affiliated Hospital of Zhengzhou University, Zhengzhou, China; 5 Department of Clinical Laboratory, The Second Affiliated Hospital of Zhengzhou University, Zhengzhou, China

**Keywords:** multiple myeloma, anemia, erythroid progenitor cells, terminal erythropoiesis, transcriptome analysis, stage specificity

## Abstract

**Background:**

Multiple myeloma (MM) associated anemia affects over 60% of patients and correlates with poor prognosis. While most studies focus on the impact of microenvironment on erythropoiesis in MM, the stage-specific erythroid cell defects and their underlying molecular mechanisms remain poorly characterized.

**Method:**

Clinical data were collected from 300 patients with newly diagnosed multiple myeloma (NDMM) and 300 healthy controls. Phenotypic and transcriptomic profiles of bone marrow early and late erythropoiesis were compared between healthy donors (HDs) and NDMM patients with anemia (NDMM-A). Furthermore, in vitro erythroid culture assays were performed to verify the intrinsic erythropoietic impairments in NDMM-A patients.

**Results:**

Primary erythropoiesis profiles from bone marrow revealed a global reduction in erythroblasts across all maturation stages, along with significantly impaired colony-forming capacity of erythroid progenitors in NDMM patients. Transcriptomic profiling identified stage-specific dysregulation in the early and late erythropoiesis of NDMM-A patients. Erythroid progenitors exhibited downregulation of cell-cycle regulators (e.g., CDC20, AURKB) and key erythropoietic transcription factors (e.g., GATA1, GFI1B), accompanied by upregulation of immune-response genes (OAS3, OAS2). Conversely, terminal erythroblasts showed upregulation of genes involved in oxidative phosphorylation and the p53 pathway. In vitro assays confirmed intrinsic defects in erythroblasts derived from NDMM patients’ CD34+ cells, including poor proliferation, increased apoptosis rate, and defective enucleation.

**Conclusion:**

These findings demonstrate that both cell-intrinsic impairments and external responses drive stage-specific erythropoietic failure in NDMM, providing new insights for therapeutic strategies.

## Introduction

Multiple myeloma (MM) is a hematologic malignancy characterized by the uncontrolled proliferation of plasma cells (Kyle & Rajkumar, 2008; Pu et al., 2024; Rajkumar, 2024). This abnormal growth disrupts normal hematopoiesis, leading to various complications, including calcium elevation, renal insufficiency, anemia, and bone disease. Among these, more than 60% of MM patients have anemia, and its severity often correlates with disease stage (Bahlis et al., 2023). The reduction in hemoglobin levels not only serves as a poor prognostic factor but is also associated with increased mortality (Liu et al., 2020; Mittelman, 2003; Musto et al., 1997; Sankaran and Weiss, 2015; Shamir et al., 2025). Understanding the cause of anemia and developing effective treatments are essential to improving the quality of life in MM patients.

The pathogenesis of anemia in MM is predominantly driven by impaired erythropoiesis. Among the various pathological factors contributing to this impairment, deficient production of erythropoietin (EPO) plays a central role. Ineffective erythropoiesis in MM is frequently attributed to insufficient EPO, a critical stimulator of red blood cell production (Deshet-Unger et al., 2016; Hiram-Bab et al., 2015). This EPO deficiency is particularly pronounced in patients with myeloma-associated renal failure (Knudsen et al., 1994). Beyond EPO deficiency, the bone marrow microenvironment in MM further disrupts erythropoiesis through multiple mechanisms. Malignant plasma cells promote apoptosis of erythroid precursors via death ligands such as FasL and TRAIL (Silvestris et al., 2002; Silvestris et al., 2001). Myeloma-secreted cytokines, including CCL3 (Han et al., 2024; Vallet et al., 2011), suppress erythropoiesis by downregulating transcription factors GATA1 and KLF1 in hematopoietic progenitor cells, impairing erythroid differentiation (Liu et al., 2020). Additionally, dysfunctional erythroblastic island macrophages overproduce IL-6, which promotes myeloma growth and suppresses erythroid differentiation (Huang et al., 2023). By far, most researches have focused on the role of the microenvironment in MM erythropoiesis. However, the stage-specific phenotypic and transcriptional features of erythropoiesis in MM-associated anemia remain largely unexplored.

Erythropoiesis is a complex, tightly controlled cellular process that comprises early erythropoiesis, terminal erythroid differentiation, and reticulocyte maturation. Many studies have reported that abnormalities at any stage of erythropoiesis can lead to abnormal erythrocyte production and subsequently cause anemia (Cazzola, 2022). Previous studies have shown that normal human erythroid progenitor cells and terminal erythroid cells display distinct stage-specific phenotypes and transcriptional regulation (An et al., 2014; Yang et al., 2024). Yet, it remains unclear what the phenotypic signatures and transcriptional regulation profiles are in early and terminal erythropoiesis in NDMM patients with anemia. This study aims to illuminate stage-specific cellular and molecular defects in early and late erythropoiesis that contribute to anemia in NDMM.

## Materials and methods

### Patients’ information and clinical data

Patients’ information and clinical data of newly diagnosed patients with multiple myeloma (NDMM) and healthy controls were collected at Zhengzhou University People’s Hospital between 2017 and 2025. The clinical characteristics of these NDMM patients are summarized in Table 1. Diagnosis of NDMM was established according to the International Myeloma Working Group criteria (Rajkumar et al., 2014). The diagnostic threshold for anemia in NDMM is a hemoglobin value > 20 g/L below the lower limit of normal, or a hemoglobin value < 100 g/L (van de Donk et al., 2021).

**TABLE 1:** General information about patients with newly diagnosed multiple myeloma.

Characteristics	Total NDMM patients (300)	Non-anemia NDMM patients (162)	Anemia NDMM patients (138)	*P*
Age [n (%)]	​	​	​	<0.001
<65 years	158 (52.7)	97 (59.9)	61 (44.2)	​
≥65 years	142 (47.3)	65 (40.1)	77 (55.8)	​
Gender [n (%)]	​	​	​	0.030
Male	190 (63.3)	110 (67.9)	80 (58.0)	​
Female	110 (36.7)	52 (32.1)	58 (42.0)	​
Type [n (%)]	​	​	​	0.079
IgG	149 (49.7)	78 (48.2)	71 (51.4)	​
IgA	70 (23.3)	38 (23.5)	32 (23.2)	​
IgD	20 (6.7)	8 (4.9)	12 (8.7)	​
Light chain	51 (17.0)	31 (19.1)	20 (14.5)	​
Non-secretory	10 (3.3)	7 (4.3)	3 (2.2)	​
DS stage [n (%)]	​	​	​	<0.001
I	142 (47.4)	142 (87.6)	0 (0.0)	​
II	58 (19.3)	10 (6.2)	48 (34.8)	​
III	100 (3.3)	10 (6.2)	90 (65.2)	​
ISS stage [n (%)]	​	​	​	<0.001
I	108 (36.0)	93 (57.4)	15 (10.9)	​
II	101 (33.7)	51 (31.5)	50 (36.2)	​
III	91 (30.3)	18 (11.1)	73 (52.9)	​
Plasma cell (x̅ ± s, %)	26.8 ± 22.5	21.6 ± 20.8	33.0 ± 23.0	<0.001
Hemoglobin (x̅ ± s, g/L)	102.1 ± 26.1	122.2 ± 14.8	78.5 ± 13.8	<0.001
RBC (x̅ ± s, ×10^12^/L)	3.3 ± 0.9	4.0 ± 0.6	2.5 ± 0.5	<0.001
MCV (x̅ ± s, pg)	95.9 ± 6.9	94.4 ± 5.5	97.6 ± 7.9	0.026
MCH (x̅ ± s, pg)	31.1 ± 2.4	31.1 ± 2.0	31.1 ± 2.8	0.802
Platelet (x̅ ± s, ×10^9^/L)	193.7 ± 120.7	219.9 ± 128.2	162.8 ± 103.6	<0.001
Leukocyte (x̅ ± s, ×10^9^/L)	5.5 ± 2.8	5.9 ± 2.9	5.0 ± 2.5	<0.001
Neutrophil (x̅ ± s, ×10^9^/L)	3.6 ± 4.3	4.0 ± 5.5	3.1 ± 2.0	0.018
Lymphocyte (x̅ ± s, ×10^9^/L)	1.5 ± 0.8	1.6 ± 0.8	1.3 ± 0.74	0.037
Monocyte (x̅ ± s, ×10^9^/L)	0.5 ± 0.8	0.5 ± 0.9	0.45 ± 0.8	0.476
Albumin (x̅ ± s, g/L)	35.8 ± 8.0	38.8 ± 6.6	32.4 ± 8.1	0.037
UREA (x̅ ± s, mmol/L)	7.9 ± 6.33	6.7 ± 5.7	9.5 ± 6.7	<0.001
CREA (x̅ ± s, μmol/L)	118.1 ± 146.5	84.3 ± 103.6	157.7 ± 176.9	<0.001
Serum calcium (x̅ ± s, mmol/L)	2.2 ± 0.3	2.3 ± 0.23	2.2 ± 0.3	<0.001
LDH [median (IQR), U/L]	186.0 (159.5–223.5)	186.0 (163.0–215.0)	183.0 (148.0–244.5)	0.145
β2-MG [median (IQR), mg/L]	3.5 (2.3–6.5)	2.6 (1.9–3.6)	6.0 (3.6–9.0)	<0.001

Table 1: The study categorized 300 NDMM, patients into a non-anemia group (hemoglobin ≥100 g/L) and an anemia group (hemoglobin <100 g/L). Abbreviations: RBC: red blood cell; MCV: mean corpuscular volume; MCH: mean corpuscular hemoglobin; CREA: creatinine; LDH: lactate dehydrogenase; DS: Durie-Salmon staging system; ISS: international staging system.

### Collection of mononuclear cells from bone marrow

This study included NDMM patients who met the International Myeloma Working Group criteria and had not received prior therapy. BM aspirates were obtained from the Zhengzhou University People’s Hospital and were residual specimens from routine clinical testing. This study was approved by the Henan Provincial People’s Hospital (Approval No. 2023–013). For mononuclear cell isolation, approximately 2–4 mL of BM aspirate was collected in EDTA-anticoagulated tubes, then diluted 2.5-fold with PBS containing 2% FBS and 2 mM EDTA (PBS/2% FBS/2 mM EDTA). The cell suspension was filtered through a 70 μm cell strainer (Biosharp, No. BS-70-CS), and the single-cell suspension was carefully layered onto 4 mL of Ficoll-Paque™ PLUS density gradient media (Cytiva, No. 17144002) in a 15 mL centrifuge tube. The cells were centrifuged at 400 g (1400 rpm) for 30 min with no brake at room temperature. Then the buffy coat (middle layer) containing mononuclear cells was collected with a transfer pipette into a 50 mL tube (up to 25 mL per tube), and washed twice with PBS/2% FBS/2 mM EDTA.

### Antibody staining and flow cytometry analysis

For staining of human primary BM erythropoiesis, 3 × 10^6^ mononuclear cells were stained with FITC-GPA (Biolegend, No.306610), APC/CH7-CD71 (BD bioscience, No.563671), PE-CD117 (BD bioscience, No.323408), APC-CD34 (BD bioscience, No.555824), Hoechst33342 (Solarbio No.C0031), PE/CF594-CD105 (BD bioscience, No.562380), PE/Cy7-IL3R (eBioscience, No.25123942), PE/Cy7-CD41a (Biolegend, No. 303718), PE/Cy7-CD45RA (Biolegend, No. 304126), then incubated on ice for 30 min in the dark. Following antibody staining and washing, cells were resuspended and subsequently stained with 7-AAD (0.5 μg/mL) on ice for 10 min in the dark. Finally, stained cells were analyzed within 1 h using an LSR Fortessa flow cytometer (Becton Dickinson).

Staining of cultured erythroblasts was performed as previously described (Yan et al., 2021). Briefly, 0.1 × 10^6^ cells were stained with FITC-GPA, APC/CH7-CD71, and PE/CF594-CD105, then incubated on ice for 30 min in the dark. For staining apoptotic cells, 0.1 × 10^6^ cells were incubated with Annexin V (Beyotime, C1062S) on ice for 20 min in the dark. For enucleation, 0.1 × 10^6^ cells were stained with Hoechst 33,342 (Beyotime, C1022) and incubated at 37 °C in the dark for 30 min.

### Colony-forming assay

A total of 5 × 10^5^ BM mononuclear cells were counted and resuspended in 1 mL IMDM supplemented with 2% FBS. From this suspension, 5 × 10^4^ mononuclear cells were taken and resuspended in 1 mL MethoCult H4330 (Stemcell #04330) medium for the CFU-E colony assay, and 1 mL MethoCult H4434 (Stemcell #04434) for the BFU-E colony assay, respectively. Cells were incubated at 37 °C in a humidified incubator with 5% CO_2_. Colonies were counted under an inverted microscope on Day 7 (CFU-E) and Day 14 (BFU-E), respectively.

### Fluorescence-activated cell sorting of human bone marrow erythroid cells

For sorting primary erythroid progenitors and terminal erythroblasts from human BM, total mononuclear cells were stained using the method of staining primary BM erythropoiesis. The sorting strategy was as follows: erythroid progenitor cells (GPA^−^CD117^+^CD34^+/−^CD45RA^−^CD41a^−^IL-3Rα^−^CD71^+^), terminal erythroblasts (GPA^+^CD71^hi^Hoechst33342^+^). Cell sorting was performed using a BD FACSAria™ Fusion cell sorter.

### Cytospin and giemsa staining

Cytospins were prepared on coated slides using 1 × 10^5^ cells with a Thermo Scientific Shandon 4 Cytospin. For Giemsa staining, Giemsa stock solution (Solarbio, G1010) was diluted 1:10 with Giemsa dilution buffer. Slides were fixed with methanol for 2 min, stained with the diluted Giemsa solution for 15 min at room temperature, and rinsed twice with distilled water. Stained cells were imaged using an inverted microscope.

### RNA-seq analysis

RNA was extracted from sorted BM primary erythroid progenitor cells and terminal erythroblasts. RNA isolation, cDNA library construction, and sequencing were performed at the Annoroad Gene Technology Institute on an Illumina NovaSeq 6000 platform using a PE150 (paired-end 150 bp) strategy, with libraries constructed from erythroid progenitors and terminal erythroblasts using the low-input Clontech SMART-Seq HT kit with Nxt HT (Takara Bio United States of America, San Jose, CA, United States of America). Raw RNA-seq data were filtered using fastp to remove low-quality reads. Gene expression was quantified with kallisto using the human reference genome hg38, and pairwise comparisons between groups were performed with DESeq2. Fold change ≥1.5 and adjusted p-value ≤0.05 were used to identify differentially expressed genes (DEGs). Principal component analysis (PCA) was conducted on log-transformed normalized counts for expressed genes. Gene Ontology (GO) enrichment analysis was performed using Metascape, with GO terms considered significant at adjusted *p*-value ≤0.01. Pathway enrichment analysis was performed using GSEA, with KEGG pathways considered significant at an adjusted *FDR* ≤0.25. The RNA-seq data supporting this study have been deposited in the NCBI Gene Expression Omnibus (GEO) database under accession number GSE301441.

### Intracellular ROS analysis

1 × 10^6^ mononuclear cells were stained using the method of staining primary BM erythropoiesis. Then the cells were stained with CellROX dye H2DCFDA (Beyotime, S0035) at a final concentration of 5 μM for 20 min at 37 °C. The cells were then washed with ice-cold PBS and resuspended in the FACS buffer (PBS +3% FBS). Finally, stained cells were analyzed within 1 h using an LSR Fortessa flow cytometer (Becton Dickinson).

### Differentiation of bone marrow CD34^+^ cells to erythroid cells

Hematopoietic stem/progenitor cells (HSPCs) were isolated using the Ultrapure CD34 Microbeads kit (Miltenyi Biotec, No. 130–100–453) according to the manufacturer’s protocol. The purified CD34^+^ cells were cultured in erythroid-inducing medium following established protocols (Qu et al., 2018; Wang S. et al., 2022). The base medium consisted of Iscove’s Modified Dulbecco’s Medium (IMDM) supplemented with 3% human AB serum, 2% human plasma, 10 μg/mL insulin, 3 IU/mL heparin, and 1% penicillin/streptomycin. The culture was divided into four phases with specific cytokine supplementation: phase 1 (Day 0–7): 200 μg/mL holo-transferrin, 3 IU/mL EPO, 10 ng/mL SCF, and 1 ng/mL IL-3; phase 2 (Day 7–11): 200 μg/mL holo-transferrin, 1 IU/mL EPO, and 10 ng/mL SCF; phase 3 (Day 11–15): 1 mg/mL holo-transferrin and 1 IU/mL EPO; and phase 4 (Day 15–20): 1 mg/mL holo-transferrin alone. Medium changes were refreshed on days 4, 7, 11, and 15. The detailed experimental scheme is illustrated in Figure 6A.

### Statistical analysis

SPSS Statistics 27.0.1 and GraphPad Prism 8 software were used for statistical analysis. FlowJo™ v10 software was used for FCS data analysis. The table data are presented as mean ± Standard Error of the Mean (SEM) or median (interquartile range, IQR), and comparisons are performed using independent-samples t-tests. Categorical variables are expressed as percentages, and the chi-squared test was used to compare them.

## Results

### Prevalence and clinical correlates of anemia in newly diagnosed multiple myeloma

**FIGURE 1:**
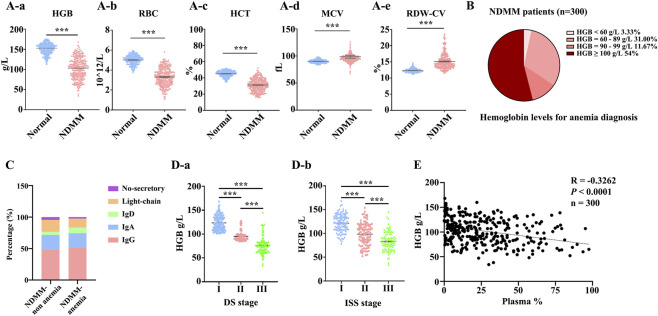
Prevalence and clinical significance of anemia in newly diagnosed multiple myeloma (NDMM) patients. **(A)** Comparative analysis of complete blood parameters between healthy controls (n = 300) and NDMM patients (n = 300), including hemoglobin (HGB) levels, red blood cell (RBC) counts, hematocrit (HCT), mean corpuscular volume (MCV), and red cell distribution width (RDW). **(B)** Proportion of NDMM patients presenting with anemia (HGB < 100 g/L) at diagnosis, categorized as mild (HGB 90–100 g/L), moderate (HGB 60–90 g/L), or severe (HGB < 60 g/L) anemia. **(C)** Comparative analysis of the distribution of myeloma subtypes between multiple myeloma patients with and without anemia. **(D)** Comparative analysis of hemoglobin levels across different disease severity groups based on the Durie-Salmon (DS) staging system **(D–a)** and the Revised International Staging System (R-ISS) **(D–b). (E)** Hemoglobin levels in NDMM patients were negatively correlated with myeloma cell infiltration in the bone marrow. ***P < 0.001.

In this study, we analyzed blood parameters from 300 healthy controls and 300 patients with newly diagnosed multiple myeloma (NDMM). Among the NDMM patients, 138 (46%) presented with anemia (hemoglobin <100 g/L) at diagnosis. Comprehensive clinical data, including demographic characteristics (age, gender), immunoglobulin isotypes, complete blood parameters, and key biochemical markers (albumin, urea, creatinine, serum calcium, lactate dehydrogenase [LDH], and β_2_-microglobulin [β_2_-MG]), are summarized in Table 1. Disease staging was assessed using both the Durie-Salmon (DS) and International Staging System (ISS) criteria. Comparative analysis revealed significantly lower hemoglobin (HGB) levels, red blood cell (RBC) counts, and hematocrit (HCT) levels in NDMM patients compared with healthy controls (Figures 1Aa–c), along with elevated mean corpuscular volume (MCV) and red cell distribution width (RDW) (Figures 1Ad,e). According to International Myeloma Working Group criteria (Rajkumar et al., 2014), the distribution of anemia severity was as follows: 11.67% (35/300) of patients had mild anemia (HGB 90–99 g/L), 31% (93/300) had moderate anemia (HGB 60–89 g/L), and 3.3% (10/300) had severe anemia (HGB <60 g/L) (Figure 1B). Among NDMM patients, the distribution of monoclonal immunoglobulin isotypes was comparable between the anemic and non-anemic groups (Figure 1C), suggesting no clear association between immunoglobulin isotype and anemia in MM patients. Notably, advanced-stage disease (Durie-Salmon stage III and R-ISS stage III) was associated with markedly lower hemoglobin levels (Figures 1Da,b), consistent with the previously reported correlation between disease progression and anemia severity (Liu et al., 2020). Correlation analysis revealed a significant inverse relationship between bone marrow plasma cell percentage and hemoglobin levels in our patient cohort (Figure 1E), supporting earlier findings that plasma cell infiltration impairs erythropoiesis in MM-associated anemia. Overall, the clinical characteristics observed in this cohort corroborate earlier findings and provide a basis for further investigation.

### The bone marrow erythroid progenitors showed quantitative and functional defects in NDMM-A patients

**FIGURE 2:**
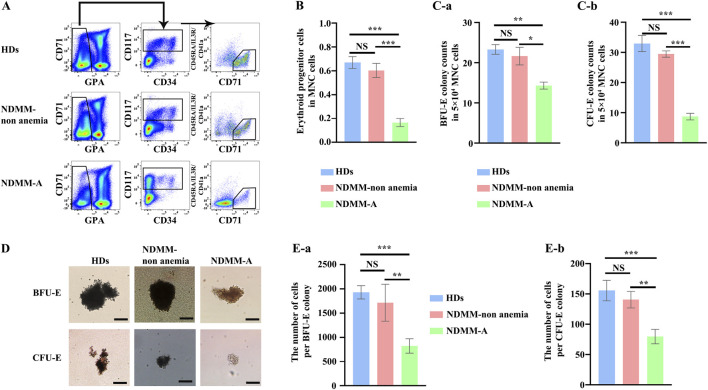
Analysis of erythroid progenitor cells in the bone marrow of controls and newly diagnosed multiple myeloma patients with anemia. (NDMM-A) **(A)** Representative flow cytometry gating strategy for identifying erythroid progenitor cells in bone marrow samples from healthy donors (HDs), NDMM patients without anemia (NDMM-non anemia), and NDMM patients with anemia (NDMM-A). **(B)** Quantification of the percentage of erythroid progenitor cells in bone marrow mononuclear cells (MNC cells) from HDs, NDMM-non anemia, and NDMM-A patients (n = 10). ***P < 0.001. **(C) (a)** The number of BFU-E colonies in 5 × 10^4^ BM cells; **(b)** The number of CFU-E colonies in 5 × 10^4^ BM cells. **(D)** Representative images of BFU-E and CFU-E colonies (scale bar: 100 μm). **(E) (a)** The number of cells in one BFU-E colony; **(b)** The number of cells in one CFU-E colony.

The erythropoiesis process comprises three main developmental stages: early-stage erythroid progenitors, including burst-forming unit-erythroid (BFU-E) and colony-forming unit-erythroid (CFU-E) cells; terminal erythroid differentiation, encompassing proerythroblasts (ProEs), basophilic erythroblasts (BasoEs), polychromatic erythroblasts (PolyEs), and orthochromatic erythroblasts (OrthoEs); and reticulocyte maturation, including reticulocytes (Retic) and mature red blood cells (RBCs) (Da Costa et al., 2020; Ebert et al., 2008; Heimpel et al., 2006). To characterize defective erythropoiesis in NDMM patients, we systematically analyzed erythroblasts at different developmental stages using the cell surface markers CD34, CD117, IL3R, CD45RA, CD41a, CD71, CD105, and GPA, as well as the nucleic acid dye Hoechst33342 and the cell viability dye 7AAD (Yan et al., 2021). Representative gating strategies of erythroid progenitor cells in mononuclear cells are shown in Figure 2A. Comparative analysis of erythroid progenitor cells revealed significant differences between HDs (0.67% ± 0.02%) and NDMM-A patients (0.17% ± 0.01%), NDMM-non anemia (0.60% ± 0.02%) and NDMM-A patients (0.17% ± 0.01%). In comparison, the HD (0.67% ± 0.02%) and NDMM-non-anemia (0.60% ± 0.02%) groups were comparable, as shown in Figure 2B. The impaired function of erythroid progenitor cells in NDMM-A was further confirmed by their reduced numbers of BFU-E and CFU-E colonies compared to those from HDs and NDMM-non anemia patients (Figures 2C, a-b). We next compared the colony-forming ability of erythroid progenitors among the NDMM-A, NDMM-non anemia, and HD groups. In the NDMM-A group, the sizes of BFU-E and CFU-E colonies were smaller than those in the control groups (Figure 2D; Supplementary Figure S1; Supplementary Figure S2). This observation was supported by quantitative analysis, which showed that the average cell number per BFU-E and CFU-E colony was significantly reduced in NDMM-A compared to both HDs and NDMM-non anemia (Figures 2E,a,b).

### Transcriptome analyses revealed both a response to external stimulus and intrinsic dysregulation in erythroid progenitors from NDMM-A patients

**FIGURE 3:**
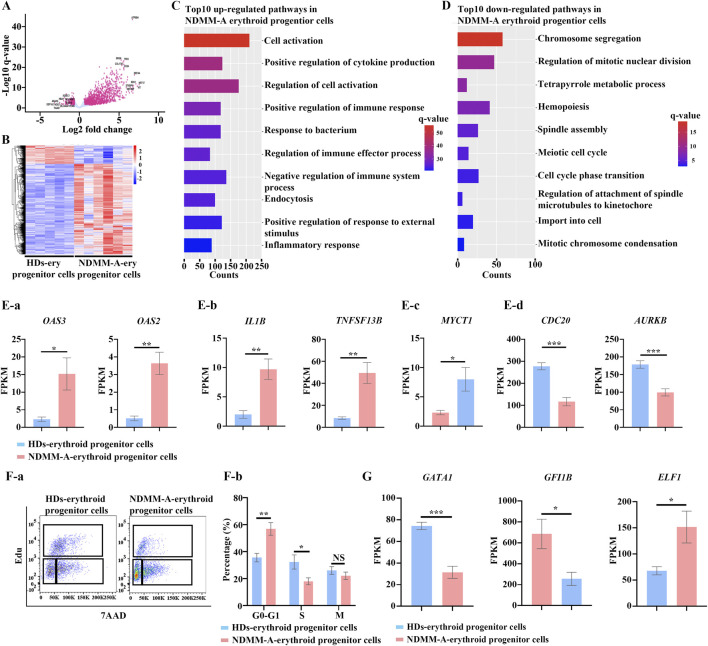
Transcriptomic analyses of bone marrow erythroid progenitor cells in healthy donors (HDs) and newly diagnosed multiple myeloma patients with anemia (NDMM-A). **(A)** Volcano plot showing differentially expressed genes in erythroid progenitors between HDs and NDMM-A. **(B)** Heatmap of DEGs in erythroid progenitor cells from HDs and NDMM-A. **(C)** The top 10 upregulated pathways in NDMM-A erythroid progenitor cells. **(D)** The top 10 downregulated pathways in NDMM-A erythroid progenitor cells. **(E)** Expression levels (FPKM) of key markers involved in regulating early-stage erythropoiesis were compared in erythroid progenitor cells from HDs and NDMM-A patients. **(F)** Cell cycle analysis of erythroid progenitor cells from HDs and NDMM-A, as measured by EdU incorporation via flow cytometry. **(G)** Expression levels (FPKM) of erythropoiesis-related regulatory markers in erythroid progenitors from HDs and NDMM patients. NS: Not Significant. *P < 0.05, **P < 0.01, ***P < 0.001.

To obtain a molecular understanding of impaired erythroid progenitor cells in NDMM, we performed transcriptomic analysis of primary BM early-stage erythroid progenitor cells (including BFU-E and CFU-E) from both normal individuals and patients with NDMM-associated anemia. The gating strategy for isolating human BM erythroid progenitors is shown in Supplementary Figure S3A. Cytospin images of sorted erythroid progenitor cells are shown in Supplementary Figure S3B. Principal component analysis (PCA) revealed separation between NDMM-A and HDs erythroid progenitor cells (Supplementary Figure S4). Approximately 13,621 genes are depicted as a volcano plot, including 1538 differentially expressed genes (DEGs) (Figure 3A). The heatmap of differentially expressed genes (DEGs) expression is shown in Figure 3B, with the DEGs listed in Supplementary Table S1. Gene ontology analysis of DEGs with increased or decreased expression in NDMM patients is shown in Figures 3C,D. Interestingly, DEGs with increased expression were not only involved in positive regulation of immune response (Figure 3C), such as *OAS3* and *OAS2* (Figures 3E–a) (Padariya et al., 2021), but also included cytokines that play a role in immune modulatory, such as as *IL1B* (Jefferies, 2019) *and TNFSF13B* (Mackay and Browning, 2002) (Figures 3E–b). We also found that *MYCT1* (Figures 3E–c), which is the key gene promoting apoptosis (Fu et al., 2018), showed higher expression in NDMM-A erythroid progenitor cells than in HDs. These changes may be attributed to differences in the BM microenvironment between NDMM-A patients and HDs. On the other hand, the DEGs with decreased expression in NDMM-As are enriched in cell cycle-related pathways, such as *CDC20* (Gao et al., 2020) and *AURKB* (Shaalan et al., 2021) (Figures 3E-d). To confirm this result, we performed an EdU incorporation assay to assess cell cycle status. As shown in Figures 3Fa–b, the percentages of cells in S-phase were much higher in HDs erythroid progenitor cells (∼30%) than in NDMM-A erythroid progenitor cells (∼18%), indicating a defective cell cycle of NDMM-A erythroid progenitor cells, which may contribute to the smaller size of colonies from NDMM-A.

In addition, erythroid-specific transcription factor *GATA1* and *GFI1B* showed decreased expression, while myeloid transcription factor *ELF1* showed increased expression (Figure 3G). *GATA1* suppression is reported to be due to secreted CCL3 in MM patients (Liu et al., 2020), which disrupts the switch and the balance between proliferation and differentiation (Munugalavadla et al., 2005)**.** *GFI1B* is reported to regulate human erythroid differentiation through Smad2/TIF1-gamma (Randrianarison-Huetz et al., 2010)*.* The downregulation of *ELF1* is necessary for erythroid differentiation (Calero-Nieto et al., 2010). Together, these findings suggest that the impaired proliferation and colony-forming ability of erythroid progenitors are both influenced by external stimulation and intrinsic dysregulation.

### A global reduction occurred in each stage of terminal erythroblasts and reticulocytes in NDMM-A patients

**FIGURE 4:**
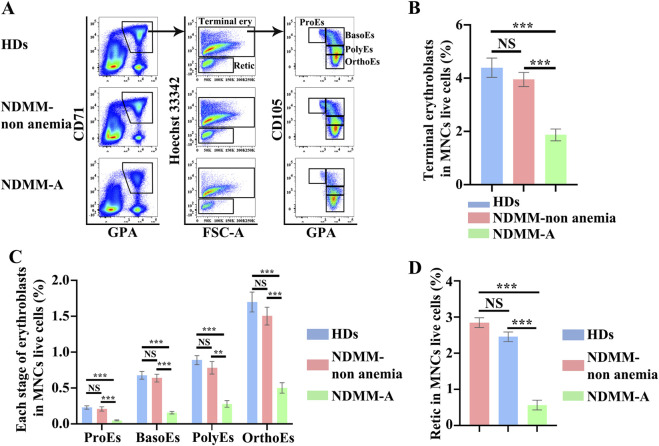
Analysis of terminal erythroblasts and reticulocytes in the bone marrow of controls and newly diagnosed multiple myeloma patients with anemia (NDMM-A). **(A)** Representative flow cytometry gating strategy for identification of terminal erythroblasts and reticulocytes in bone marrow samples from HDs, NDMM-non anemia, and NDMM-A patients. **(B,C)** Frequency of total terminal erythroblasts and their subpopulations: proerythroblasts ProEs, basophilic erythroblasts BasoEs, polychromatic erythroblasts PolyEs, and orthochromatic erythroblasts OrthoEs, in bone marrow mononuclear cells from HDs, NDMM-non anemia, and NDMM-A patients (n = 10). **(D)** Frequency of reticulocytes in bone marrow mononuclear cells (n = 10). NS: Not Significant, **P < 0.01, ***P < 0.001.

We further systematically characterized terminal erythroblasts and reticulocytes by flow cytometry using CD71, Hoechst33342, CD105, and GPA markers (Yan et al., 2021). Initial gating of CD71^+^ and GPA^+^ populations consists of two distinct subsets: nucleated erythroblasts (Hoechst33342^+^) and reticulocytes (Hoechst33342^-^), shown as Figure 4A. Within the terminal erythroblasts (CD71^+^GPA^+^Hoechst33342^+^) population, which were further separated into four precursor populations based on CD105 and GPA expression: GPA^low^CD105^high^ as ProEs, GPA^high^CD105^high/int^ as BasoEs, GPA^high^CD105^low^ as PolyEs, and GPA^high^CD105^-^ as OrthoEs (Figure 4A). Quantitative analysis revealed severe terminal erythroblast depletion in NDMM-A patients compared to HDs and NDMM-non anemia (Figure 4B). Stage-specific analysis demonstrated a decrease at each stage of terminal erythropoiesis in NDMM-A patients (Figure 4C). Furthermore, the percentage of reticulocytes in BM mononuclear cells (CD71^+^GPA^+^Hoechst33342^-^) was also significantly decreased in NDMM-A patients compared to HDs and NDMM-non anemia (Figure 4D). These findings collectively highlight overall suppression at all stages of terminal erythropoiesis in NDMM-A patients.

### Genes involved in the positive regulation of p53 signal and oxidative phosphorylation (OXPHOS) showed increased expression in terminal erythroblasts of NDMM-A patients

**FIGURE 5:**
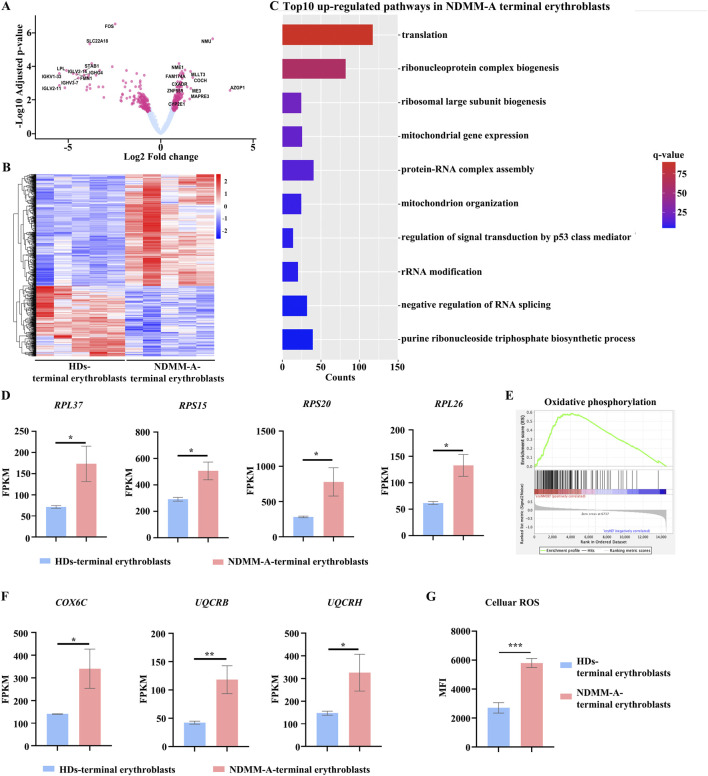
Transcriptomic analyses of bone marrow terminal erythroblasts in healthy donors (HDs) and newly diagnosed multiple myeloma patients with anemia (NDMM-A). **(A)** Volcano plot showing differentially expressed genes in terminal erythroblasts between HDs and NDMM-A. **(B)** Heatmap of DEGs across samples from HDs and NDMM-A. **(C)** The top 10 upregulated pathways in NDMM-A terminal erythroblasts. **(D)** Expression levels (FPKM) of key genes involved in the p53 signaling pathway in terminal erythroblasts from HDs and NDMM-A. **(E)** Gene Set Enrichment Analysis (GSEA) plots demonstrating significant enrichment of the oxidative phosphorylation gene set in NDMM-A terminal erythroblasts compared to HDs. **(F)** Expression levels (FPKM) of key genes involved in oxidative phosphorylation in terminal erythroblasts from HDs and NDMM-A. **(G)** Intracellular reactive oxygen species (ROS) levels in primary bone marrow terminal erythroblasts from HDs and NDMM-A, as measured by H_2_DCFDA staining. *P < 0.05, **P < 0.01, ***P < 0.001.

To understand the reduction of terminal erythroblasts in NDMM-A patients, we also performed comparative transcriptomic analysis between terminal erythroblasts (including ProEs, BasoEs, PolyEs, and OrthoEs) isolated from NDMM-A and HDs’ bone marrow. The gating strategy for isolating human BM terminal erythroblasts is shown in Supplementary Figure S3A. Cytospin images of sorted terminal erythroblasts are shown in Supplementary Figure S3B. Principal component analysis (PCA) revealed separation between NDMM-A and HDs terminal erythroblasts (Supplementary Figure S5). A volcano plot shows approximately 13,724 genes, including 638 DEGs (Figure 5A). The heatmap of DEGs expression is shown in Figure 5B, with the DEGs listed in Supplementary Table S2. GO enrichment of DEGs with increased expression in NDMM-A patients revealed dysregulation of translation, and positive regulation of signal transduction by p53 class mediator (Figure 5C). Interestingly, the expression levels of ribosome protein genes, including *RPL37*, *RPS15*, *RPS20*, and *RPL26*, which have been shown to lead to p53 stabilization and induce cell death and cell cycle arrest (Daftuar et al., 2013; Zhang et al., 2010), were all significantly upregulated in NDMM-A terminal erythroblasts (Figure 5D). Gene set enrichment analysis (GSEA) further revealed upregulation of genes involved in oxidative phosphorylation (OXPHOS) in NDMM-A terminal erythroblasts (Figure 5E). Notably, expression levels of mitochondrial apoptosis marker, cytochrome c oxidase subunit Vic (*COX6C*) (Wang C. et al., 2022), and also the expression of genes involved in the function of mitochondrial complex III, ubiquinol cytochrome c reductase binding protein (*UQCRB*) (Jung et al., 2010), and ubiquinol cytochrome c reductase hinge (*UQCRH*) (Gao et al., 2016) were significantly elevated in NDMM-A terminal erythroblasts with all fold change > 1 (Figure 5F). These components are known to promote mitochondrial reactive oxygen species (ROS) production. To validate these findings, we quantified intracellular ROS levels using DCFH-DA. Consistent with transcriptomic data, flow cytometric analysis confirmed elevated total ROS (Figure 5G) in NDMM-A terminal erythroblasts compared to HDs. Taken together, our results indicated that decreased terminal erythroblasts in NDMM-A patients were due to mitochondrial OXPHOS hyperactivation and p53 pathway induction, ultimately leading to ROS-mediated cell death.

### Erythroid cells differentiated from BM CD34^+^ cells of NDMM-A patients exhibited impaired proliferation and enucleation

**FIGURE 6:**
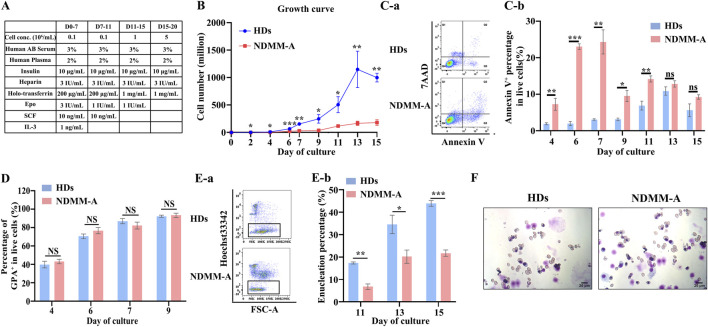
Impaired proliferation and enucleation of erythroid cells derived from newly diagnosed multiple myeloma patients with anemia (NDMM-A)-CD34^+^ cells *in vitro*. **(A)** Schematic diagram of the three-phase *in vitro* erythroid differentiation culture system. **(B)** Growth kinetics of erythroid cells derived from CD34^+^ hematopoietic stem/progenitor cells of HDs and NDMM-A patients during *in vitro* differentiation. **(C)** Apoptosis analysis during erythroid differentiation. **(C-a)** Representative flow cytometry plots of Annexin V/7AAD staining on cultured day 7. **(C-b)** Quantitative analysis of Annexin V^+^ rates (early and late apoptotic cells) at the indicated time points. **(D)** Quantitative analysis of GPA^+^ percentage on different culture days. **(E-a)** Representative flow cytometry profiles of Hoechst33342 staining on cultured day 15; **(E-b)** Quantitative assessment of enucleation rates at different culture time points. **(F)** Representative images showing morphological features of erythroblasts on cultured day 15. Scale bar: 20 μm.

Previous studies have shown that extrinsic factors can influence erythropoiesis in MM patients with anemia (Bouchnita et al., 2016). While it remains unclear whether the intrinsic erythroid differentiation is also compromised. Our transcriptomic analyses revealed both microenvironmental and intrinsic dysregulation, including decreased expression of key erythroid regulators, such as *GATA1* and *GFI1B*. To confirm the intrinsic erythroid defects without the influence of the BM microenvironment, we compared the *in vitro* erythroid differentiation potential of BM HSPCs-CD34^+^ from anemic NDMM patients (NDMM-A-CD34^+^) to those from healthy controls (HDs-CD34^+^) using a three-phase erythroid differentiation protocol (Qu et al., 2018; Wang S. et al., 2022), as detailed in Figure 6A. Cell growth was first monitored, and the growth curve is presented in Figure 6B. Under the same experimental conditions, HDs-CD34^+^cells generated 6 times more erythroid cells than NDMM-A-CD34^+^cells on culture day 15. To explore the underlying cause of this defective cell growth, we checked erythroblast apoptosis on different culture days. Representative flow cytometry plots of 7-AAD and Annexin V double staining on day 7 are shown in Figures 6C–a. Quantitative analysis revealed that significantly increased apoptosis in NDMM-A-CD34^+^ derived erythroid cells on days 4, 6, 7, 9, and 11 compared to HDs (Figures 6C,b). Next, we evaluated erythroid differentiation using GPA. The percentages of terminal erythroblasts were comparable between groups on days 4, 6, 7, and 9, indicating that the differentiation of erythroid cells was similar between HDs and from NDMM-A-CD34^+^ (Figure 6D).

Enucleation is the final step in erythropoiesis, and enucleation rates have been reported to correlate with disease status in MDS (An et al., 2023). We also assessed this process using Hoechst33342 staining. Representative profiles are shown in Figures 6E–a. Quantitative analysis revealed a significantly lower enucleation rate in NDMM-A-CD34^+^ derived OrthoEs than in controls (Figures 6E–b). Consistent with these findings, morphological analysis on day 15 further supported defective erythroid maturation in NDMM-A patients (Figure 6F). Together, these data demonstrate that intrinsic impairments in erythroid development, particularly in survival and enucleation, contribute to anemia in NDMM patients.

## Discussion

The mechanisms underlying anemia in newly diagnosed multiple myeloma (NDMM) remain poorly explored, despite its high prevalence and clinical consequences. Here, we integrated clinical, functional, and transcriptomic analyses to explain these mechanisms. Compared with healthy donors and NDMM-non anemia patients, NDMM patients with anemia exhibited reduced erythroid progenitors, terminal erythroblasts, and reticulocytes, along with impaired colony-forming capacity. Stage-specific transcriptomic dysregulation was observed; erythroid progenitor cells showed downregulation of cell cycle and key erythropoietic regulators, while terminal erythroblasts displayed activated oxidative phosphorylation and P53 pathways, accompanied by elevated ROS. *In vitro* culture of CD34^+^ cells further confirmed intrinsic erythroid defects, including poor proliferation, increased apoptosis, and defective enucleation in NDMM-A-derived erythroblasts. These results reveal stage-specific and cell-intrinsic erythropoietic impairment in MM, providing a transcriptomic resource for future research.

To investigate impaired erythropoiesis in NDMM-associated anemia, we first analyzed BM erythroid progenitor cells (BFU-E and CFU-E) from NDMM-A patients and controls. Flow cytometry analysis revealed a significant decrease in the proportion of erythroid progenitors in NDMM-A patients. This reduction was further confirmed by a decreased number of BFU-E and CFU-E colonies in methylcellulose assays. Moreover, the colony-forming capacity of these progenitors was compromised, as evidenced by a reduction in the number of cells per colony. These results indicate not only a quantitative decrease in erythroid progenitors in NDMM-A BM, but also an intrinsic functional impairment in their proliferative potential under standard culture conditions containing 3 U/ml rhEPO in methylcellulose medium. This finding aligns with earlier studies demonstrating a reduced number or reduced sensitivity of EPO receptors (EPOR) on BFU-E and CFU-E in myeloma patients, which can be partially restored by high concentrations of rhEPO (Aoki et al., 1992). Reduced EPOR sensitivity may be linked to overproduction of cytokines in the BM microenvironment of MM patients. For instance, elevated serum levels of cytokines such as TNF-α (Jasrotia et al., 2020) in NDMM patients can suppress erythropoiesis by downregulating EPOR signaling and key erythroid transcription factors (GATA-1, FOG-1) (Buck et al., 2008). Consistently, our RNA-seq data of erythroid progenitor cells further confirmed the downregulation of *EPOR* expression (data not shown). EPOR downregulation in MM provides a mechanistic explanation for the observed erythroid progenitor cell dysfunction under physiological EPO levels *in vitro*. The impaired colony-forming ability of erythroid progenitors seems at odds with clinical observations that rh-EPO is effective in improving anemia in multiple myeloma patients. Actually, the standard therapeutic doses of rhEPO (Terpos et al., 2015) (e.g., 30,000–40,000 U/week) far exceed the concentration used in colony assays (3 U/ml) and physiological status. High-dose rhEPO application likely overcomes the observed progenitor defect by supersaturating the available EPOR, thereby amplifying downstream survival and proliferative signals even in a setting of reduced receptor sensitivity or number.

Transcriptomic analysis revealed stage-specific regulation in early- and late-stage erythropoiesis in NDMM-A patients. Transcriptomic profiling of erythroid progenitors from NDMM-A revealed significant upregulation of the immune response pathway, including *OAS3* and *OAS2*, compared with HDs. Additionally, elevated *IL1B* expression was observed in NDMM-A patients, which is consistent with previous reports that plasma IL-1β level is correlated with the risk of cachexia in patients with MM (Mazurek et al., 2024). It has been reported that myeloma-cell-secreted type I interferon (IFN) induces the expression of IFN-stimulated genes such as *IFIT1, IFIT3, OAS2, and OAS3*, which are critically involved in innate antiviral responses (Kawano et al., 2018), and our data showed consistent results. Furthermore, type I IFN production, particularly IFNα, has been shown to suppress erythropoiesis by negatively modulating erythropoietin (EPO) signaling (Han et al., 2024). These observations collectively suggest a model in which tumor-mediated induction of type I IFN signaling contributes to anemia. The upregulation of IFN-responsive genes (e.g., *OAS3, OAS2*) and the cytokine *IL1B* in erythroblasts of NDMM-A patients may reflect a response to chronic inflammatory signals emanated from the malignant microenvironment. These alterations could impair erythroid maturation and amplify inflammatory cascades, potentially facilitating immune evasion by myeloma cells, thereby confirming the microenvironmental effects of erythropoiesis defects in MM. In addition, our *ex vivo* experiments indicated normal erythroid differentiation but a significantly reduced enucleation rate, which differs from the *in vivo* erythropoietic phenotype observed in MM patients. This discrepancy between *in vivo* and *ex vivo* findings further supports previous reports that the bone marrow microenvironment plays an essential role in the erythropoietic defects observed in anemic MM patients.

NDMM-A BM terminal erythroblasts indicated upregulation of genes involved in translation, p53-mediated pathway, and OXPHOS in NDMM-A patients. Elevated ROS levels were also confirmed in terminal erythroblasts from NDMM-A BM. The results indicated that p53 activation is an intrinsic response to the elevated oxidative environment, ultimately leading to a decrease in erythroid precursors.

*In vitro* studies are generated under controlled culture conditions with optimal levels of EPO and other erythroid-supportive cytokines, effectively decouple the intrinsic erythropoiesis from extrinsic inhibitory factors. The inherent defects are confirmed by *in vitro* differentiation assays, which indicated elevated apoptosis and defective enucleation in erythroblasts derived from NDMM-A CD34^+^ cells. The persistence of these defects in this context provides evidence for a cell-autonomous defect within the erythroid lineage of MM patients that is independent of the bone marrow microenvironment. However, the underlying mechanisms remain to be fully elucidated.

Together, anemia in MM patients is affected by both the microenvironment of erythropoiesis and intrinsic dysregulation within erythroblasts. These findings offer valuable insights for clinical intervention.

## Data Availability

The original contributions presented in the study are included in the article/Supplementary Material, further inquiries can be directed to the corresponding authors.

## Supplementary material

SUPPLEMENTARY FIGURE S1
 Representative images of BFU-E and CFU-E colonies derived from bone marrow (BM) cells of healthy controls and NDMM patients with anemia. Scale bar: 100 μm.

SUPPLEMENTARY FIGURE S2
 (A) Flow cytometry gating strategy for isolating BM erythroid progenitor cells and terminal erythroblasts from mononuclear cells in human bone marrow. (B) Representative images of sorted erythroid progenitors and terminal erythroblasts from healthy controls and NDMM patients with anemia. Scale bar: 20 μm.

SUPPLEMENTARY TABLE S1
Complete list of differentially expressed genes (DEGs) in BM erythroid progenitor cells from healthy controls and NDMM patients with anemia.

SUPPLEMENTARY TABLE S1
Complete list of differentially expressed genes (DEGs) in BM terminal erythroblasts from healthy controls and NDMM patients with anemia.

## References

[B1] An X. Schulz V. P. Li J. Wu K. Liu J. Xue F. (2014). Global transcriptome analyses of human and murine terminal erythroid differentiation. Blood 123, 3466–3477. 10.1182/blood-2014-01-548305 24637361 PMC4041167

[B2] An C. Xue F. Sun L. Han H. Zhang Y. Hu Y. (2023). The impact of erythroblast enucleation efficiency on the severity of anemia in patients with myelodysplastic syndrome. Cell Commun. Signal 21, 332. 10.1186/s12964-023-01353-4 37986081 PMC10658927

[B3] Aoki I. Nishijima K. Homori M. Nakahara K. Higashi K. Ishikawa K. (1992). Responsiveness of bone marrow erythroid progenitors (CFU-E and BFU-E) to recombinant human erythropoietin (rh-Ep) *in vitro* in multiple myeloma. Br. J. Haematol. 81, 463–469. 10.1111/j.1365-2141.1992.tb02976.x 1390230

[B4] Bahlis N. J. Costello C. L. Raje N. S. Levy M. Y. Dholaria B. Solh M. (2023). Elranatamab in relapsed or refractory multiple myeloma: the MagnetisMM-1 phase 1 trial. Nat. Med. 29, 2570–2576. 10.1038/s41591-023-02589-w 37783970 PMC10579053

[B5] Bouchnita A. Eymard N. Moyo T. K. Koury M. J. Volpert V. (2016). Bone marrow infiltration by multiple myeloma causes anemia by reversible disruption of erythropoiesis. Am. J. Hematol. 91, 371–378. 10.1002/ajh.24291 26749142

[B6] Buck I. Morceau F. Cristofanon S. Heintz C. Chateauvieux S. Reuter S. (2008). Tumor necrosis factor alpha inhibits erythroid differentiation in human erythropoietin-dependent cells involving p38 MAPK pathway, GATA-1 and FOG-1 downregulation and GATA-2 upregulation. Biochem. Pharmacol. 76, 1229–1239. 10.1016/j.bcp.2008.08.025 18805401

[B7] Calero-Nieto F. J. Wood A. D. Wilson N. K. Kinston S. Landry J. R. Göttgens B. (2010). Transcriptional regulation of Elf-1: locus-wide analysis reveals four distinct promoters, a tissue-specific enhancer, control by PU.1 and the importance of Elf-1 downregulation for erythroid maturation. Nucleic Acids Res. 38, 6363–6374. 10.1093/nar/gkq490 20525788 PMC2965225

[B8] Cazzola M. (2022). Ineffective erythropoiesis and its treatment. Blood 139, 2460–2470. 10.1182/blood.2021011045 34932791

[B9] Da Costa L. Leblanc T. Mohandas N. (2020). Diamond-Blackfan anemia. Blood 136, 1262–1273. 10.1182/blood.2019000947 32702755 PMC7483438

[B10] Daftuar L. Zhu Y. Jacq X. Prives C. (2013). Ribosomal proteins RPL37, RPS15 and RPS20 regulate the Mdm2-p53-MdmX network. PLoS One 8, e68667. 10.1371/journal.pone.0068667 23874713 PMC3713000

[B11] Deshet-Unger N. Hiram-Bab S. Haim-Ohana Y. Mittelman M. Gabet Y. Neumann D. (2016). Erythropoietin treatment in murine multiple myeloma: immune gain and bone loss. Sci. Rep. 6, 30998. 10.1038/srep30998 27481313 PMC4969594

[B12] Ebert B. L. Galili N. Tamayo P. Bosco J. Mak R. Pretz J. (2008). An erythroid differentiation signature predicts response to lenalidomide in myelodysplastic syndrome. PLoS Med. 5, e35. 10.1371/journal.pmed.0050035 18271621 PMC2235894

[B13] Fu S. Fu Y. Chen F. Hu Y. Quan B. Zhang J. (2018). Overexpression of MYCT1 inhibits proliferation and induces apoptosis in human acute myeloid leukemia HL-60 and KG-1a cells *in vitro* and *in vivo* . Front. Pharmacol. 9, 1045. 10.3389/fphar.2018.01045 30283340 PMC6157318

[B14] Gao F. Liu Q. Li G. Dong F. Qiu M. Lv X. (2016). Identification of ubiquinol cytochrome c reductase hinge (UQCRH) as a potential diagnostic biomarker for lung adenocarcinoma. Open Biol. 6, 150256. 10.1098/rsob.150256 27358292 PMC4929934

[B50] Gao Y. Wen P. Chen B. Hu G. Wu L. Xu A. (2020). Downregulation of CDC20 increases radiosensitivity through Mcl-1/p-Chk1-mediated DNA damage and apoptosis in tumor cells. Int. J. Mol. Sci. 21 (18), 6692. 10.3390/ijms21186692 32932732 PMC7555290

[B15] Han Y. Gao C. Liu Y. Zhang H. Wang S. Zhao H. (2024). Hemolysis-driven IFNα production impairs erythropoiesis by negatively regulating EPO signaling in sickle cell disease. Blood 143, 1018–1031. 10.1182/blood.2023021658 38127913 PMC10950476

[B16] Heimpel H. Schwarz K. Ebnöther M. Goede J. S. Heydrich D. Kamp T. (2006). Congenital dyserythropoietic anemia type I (CDA I): molecular genetics, clinical appearance, and prognosis based on long-term observation. Blood 107, 334–340. 10.1182/blood-2005-01-0421 16141353

[B17] Hiram-Bab S. Liron T. Deshet-Unger N. Mittelman M. Gassmann M. Rauner M. (2015). Erythropoietin directly stimulates osteoclast precursors and induces bone loss. Faseb J. 29, 1890–1900. 10.1096/fj.14-259085 25630969

[B18] Huang H. Yu P. Y. Wei C. Li Y. W. Liang L. J. Liu Y. Z. (2023). Regulatory effect and mechanism of erythroblastic island macrophages on anemia in patients with newly diagnosed multiple myeloma. J. Inflamm. Res. 16, 2585–2594. 10.2147/jir.S413044 37350774 PMC10284299

[B49] Jasrotia S. Gupta R. Sharma A. Halder A. Kumar L. (2020). Cytokine profile in multiple myeloma. Cytokine 136, 155271. 10.1016/j.cyto.2020.155271 32916474

[B19] Jefferies C. A. (2019). Regulating IRFs in IFN driven disease. Front. Immunol. 10, 325. 10.3389/fimmu.2019.00325 30984161 PMC6449421

[B20] Jung H. J. Shim J. S. Lee J. Song Y. M. Park K. C. Choi S. H. (2010). Terpestacin inhibits tumor angiogenesis by targeting UQCRB of mitochondrial complex III and suppressing hypoxia-induced reactive oxygen species production and cellular oxygen sensing. J. Biol. Chem. 285, 11584–11595. 10.1074/jbc.M109.087809 20145250 PMC2857036

[B21] Kawano Y. Zavidij O. Park J. Moschetta M. Kokubun K. Mouhieddine T. H. (2018). Blocking IFNAR1 inhibits multiple myeloma-driven treg expansion and immunosuppression. J. Clin. Invest 128, 2487–2499. 10.1172/jci88169 29558366 PMC5983341

[B22] Knudsen L. M. Hippe E. Hjorth M. Holmberg E. Westin J. (1994). Renal function in newly diagnosed multiple myeloma--a demographic study of 1353 patients. The nordic myeloma study group. Eur. J. Haematol. 53, 207–212. 10.1111/j.1600-0609.1994.tb00190.x 7957804

[B23] Kyle R. A. Rajkumar S. V. (2008). Multiples myeloma. Blood 111, 2962–2972. 10.1182/blood-2007-10-078022 18332230 PMC2265446

[B24] Liu L. Yu Z. Cheng H. Mao X. Sui W. Deng S. (2020). Multiple myeloma hinders erythropoiesis and causes anaemia owing to high levels of CCL3 in the bone marrow microenvironment. Sci. Rep. 10, 20508. 10.1038/s41598-020-77450-y 33239656 PMC7689499

[B25] Mackay F. Browning J. L. (2002). BAFF: a fundamental survival factor for B cells. Nat. Rev. Immunol. 2, 465–475. 10.1038/nri844 12094221

[B26] Mazurek M. Szudy-Szczyrek A. Homa-Mlak I. Hus M. Małecka-Massalska T. Mlak R. (2024). IL1B polymorphism (rs1143634) and IL-1β plasma concentration as predictors of nutritional disorders and prognostic factors in multiple myeloma patients. Cancers (Basel) 16doi, 1263. 10.3390/cancers16071263 38610941 PMC11011170

[B27] Mittelman M. (2003). The implications of anemia in multiple myeloma. Clin. Lymphoma 4 (Suppl. 1), S23–S29. 10.3816/clm.2003.s.005 14556675

[B28] Munugalavadla V. Dore L. C. Tan B. L. Hong L. Vishnu M. Weiss M. J. (2005). Repression of c-kit and its downstream substrates by GATA-1 inhibits cell proliferation during erythroid maturation. Mol. Cell Biol. 25, 6747–6759. 10.1128/mcb.25.15.6747-6759.2005 16024808 PMC1190349

[B29] Musto P. Falcone A. D'Arena G. Scalzulli P. R. Matera R. Minervini M. M. (1997). Clinical results of recombinant erythropoietin in transfusion-dependent patients with refractory multiple myeloma: role of cytokines and monitoring of erythropoiesis. Eur. J. Haematol. 58, 314–319. 10.1111/j.1600-0609.1997.tb01677.x 9222286

[B30] Padariya M. Sznarkowska A. Kote S. Gómez-Herranz M. Mikac S. Pilch M. (2021). Functional interfaces, biological pathways, and regulations of interferon-related DNA damage resistance signature (IRDS) genes. Biomol. 11, 622. 10.3390/biom11050622 33922087 PMC8143464

[B31] Pu J. Liu T. Wang X. Sharma A. Schmidt-Wolf I. G. H. Jiang L. (2024). Exploring the role of histone deacetylase and histone deacetylase inhibitors in the context of multiple myeloma: mechanisms, therapeutic implications, and future perspectives. Exp. Hematol. Oncol. 13, 45. 10.1186/s40164-024-00507-5 38654286 PMC11040994

[B32] Qu X. Zhang S. Wang S. Wang Y. Li W. Huang Y. (2018). TET2 deficiency leads to stem cell factor-dependent clonal expansion of dysfunctional erythroid progenitors. Blood 132, 2406–2417. 10.1182/blood-2018-05-853291 30254129 PMC6265651

[B33] Rajkumar S. V. (2024). Multiple myeloma: 2024 update on diagnosis, risk-stratification, and management. Am. J. Hematol. 99, 1802–1824. 10.1002/ajh.27422 38943315 PMC11404783

[B34] Rajkumar S. V. Dimopoulos M. A. Palumbo A. Blade J. Merlini G. Mateos M. V. (2014). International myeloma working group updated criteria for the diagnosis of multiple myeloma. Lancet Oncol. 15, e538–e548. 10.1016/s1470-2045(14)70442-5 25439696

[B35] Randrianarison-Huetz V. Laurent B. Bardet V. Blobe G. C. Huetz F. Duménil D. (2010). Gfi-1B controls human erythroid and megakaryocytic differentiation by regulating TGF-beta signaling at the bipotent erythro-megakaryocytic progenitor stage. Blood 115, 2784–2795. 10.1182/blood-2009-09-241752 20124515

[B36] Sankaran V. G. Weiss M. J. (2015). Anemia: progress in molecular mechanisms and therapies. Nat. Med. 21, 221–230. 10.1038/nm.3814 25742458 PMC4452951

[B37] Shaalan A. K. Teshima T. H. N. Tucker A. S. Proctor G. B. (2021). Inhibition of Aurora kinase B activity disrupts development and differentiation of salivary glands. Cell Death Discov. 7, 16. 10.1038/s41420-020-00393-w 33462217 PMC7814035

[B38] Shamir G. Rouvio O. Reiner-Benaim A. (2025). Prognostic and biological characteristics associated with multiple myeloma presenting only with anemia. Haematologica 110, 2469–2474. 10.3324/haematol.2024.286457 40304075 PMC12485322

[B39] Silvestris F. Tucci M. Cafforio P. Dammacco F. (2001). Fas-L up-regulation by highly malignant myeloma plasma cells: role in the pathogenesis of anemia and disease progression. Blood 97, 1155–1164. 10.1182/blood.v97.5.1155 11222356

[B40] Silvestris F. Cafforio P. Tucci M. Dammacco F. (2002). Negative regulation of erythroblast maturation by Fas-L(+)/TRAIL(+) highly malignant plasma cells: a major pathogenetic mechanism of anemia in multiple myeloma. Blood 99, 1305–1313. 10.1182/blood.v99.4.1305 11830480

[B41] Terpos E. Kleber M. Engelhardt M. Zweegman S. Gay F. Kastritis E. (2015). European myeloma network guidelines for the management of multiple myeloma-related complications. Haematologica 100, 1254–1266. 10.3324/haematol.2014.117176 26432383 PMC4591757

[B48] Vallet S. Pozzi S. Patel K. Vaghela N. Fulciniti M. T. Veiby P. (2011). A novel role for CCL3 (MIP-1α) in myeloma-induced bone disease via osteocalcin downregulation and inhibition of osteoblast function. Leukemia 25, 1174–1181. 10.1038/leu.2011.43 21403648 PMC4142423

[B42] van de Donk N. Pawlyn C. Yong K. L. (2021). Multiple myeloma. Lancet 397, 410–427. 10.1016/s0140-6736(21)00135-5 33516340

[B43] Wang C. Lv J. Xue C. Li J. Liu Y. Xu D. (2022). Novel role of COX6c in the regulation of oxidative phosphorylation and diseases. Cell Death Discov. 8, 336. 10.1038/s41420-022-01130-1 35879322 PMC9314418

[B44] Wang S. Zhao H. Zhang H. Gao C. Guo X. Chen L. (2022). Analyses of erythropoiesis from embryonic stem cell-CD34(+) and cord blood-CD34(+) cells reveal mechanisms for defective expansion and enucleation of embryomic stem cell-erythroid cells. J. Cell Mol. Med. 26, 2404–2416. 10.1111/jcmm.17263 35249258 PMC8995447

[B45] Yan H. Ali A. Blanc L. Narla A. Lane J. M. Gao E. (2021). Comprehensive phenotyping of erythropoiesis in human bone marrow: evaluation of normal and ineffective erythropoiesis. Am. J. Hematol. 96, 1064–1076. 10.1002/ajh.26247 34021930 PMC8355124

[B46] Yang Q. Chen L. Zhang H. Li M. Sun L. Wu X. (2024). DNMT1 regulates human erythropoiesis by modulating cell cycle and endoplasmic reticulum stress in a stage-specific manner. Cell Death Differ. 31, 999–1012. 10.1038/s41418-024-01305-6 38719927 PMC11303534

[B47] Zhang Y. Wang J. Yuan Y. Zhang W. Guan W. Wu Z. (2010). Negative regulation of HDM2 to attenuate p53 degradation by ribosomal protein L26. Nucleic Acids Res. 38, 6544–6554. 10.1093/nar/gkq536 20542919 PMC2965247

