# Supplementary figures and images for: Stage-specific disruption of erythropoiesis leads to anemia in newly diagnosed multiple myeloma patients
Source: Front Cell Dev Biol. 2026 Jun 10;14:1718025. doi: 10.3389/fcell.2026.1718025 (PMC13291475; doi:10.3389/fcell.2026.1718025)

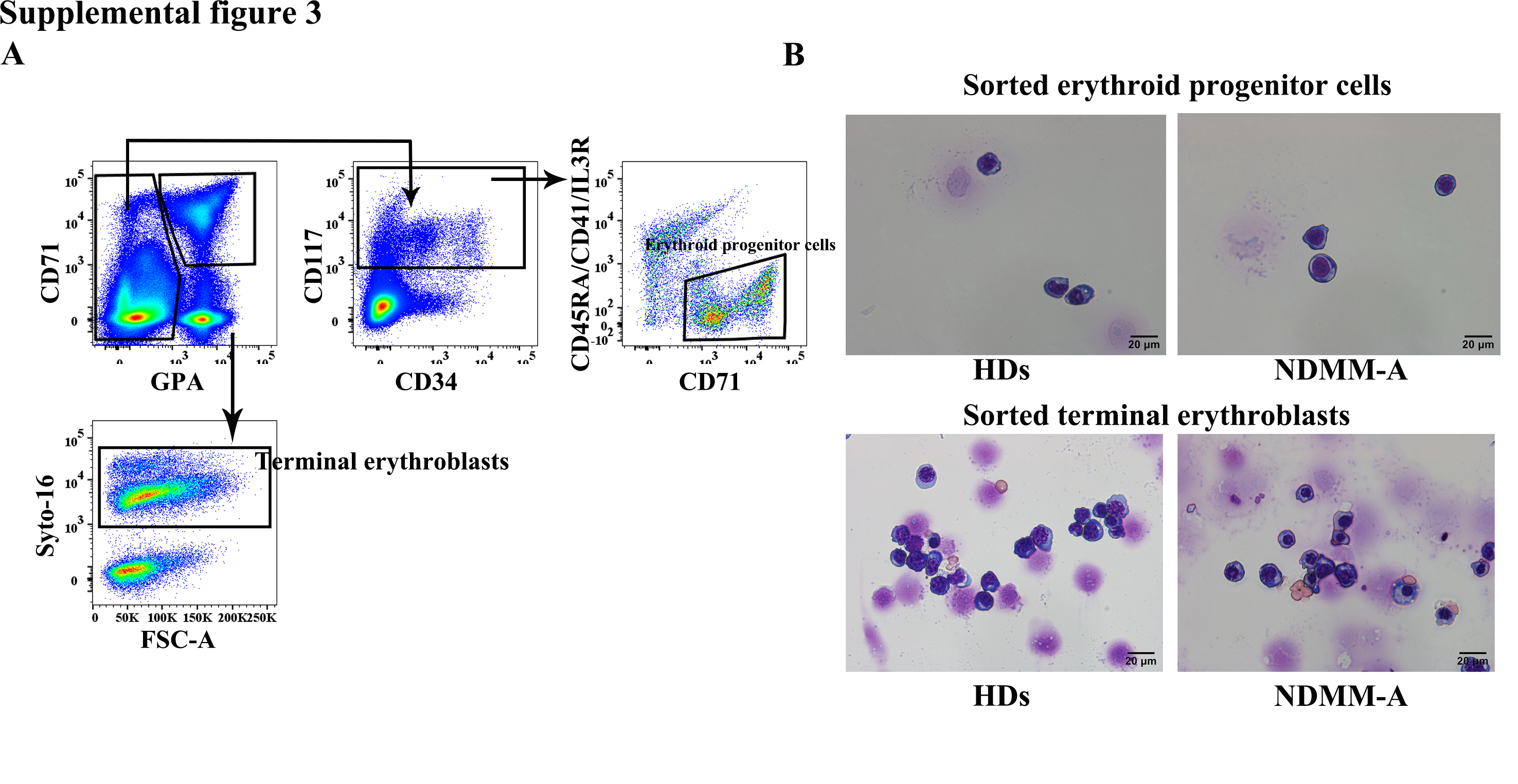

Supplement: Supplementary file 2 [file Image3.tif]

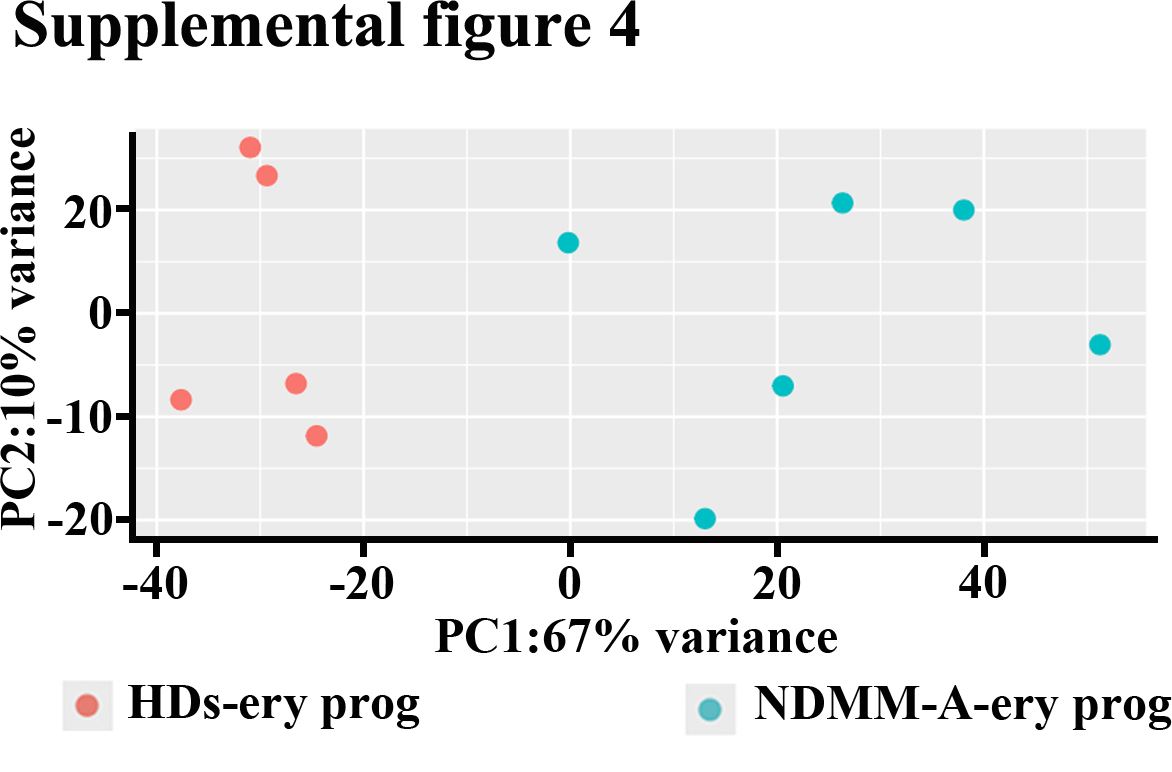

Supplement: Supplementary file 3 [file Image4.tif]

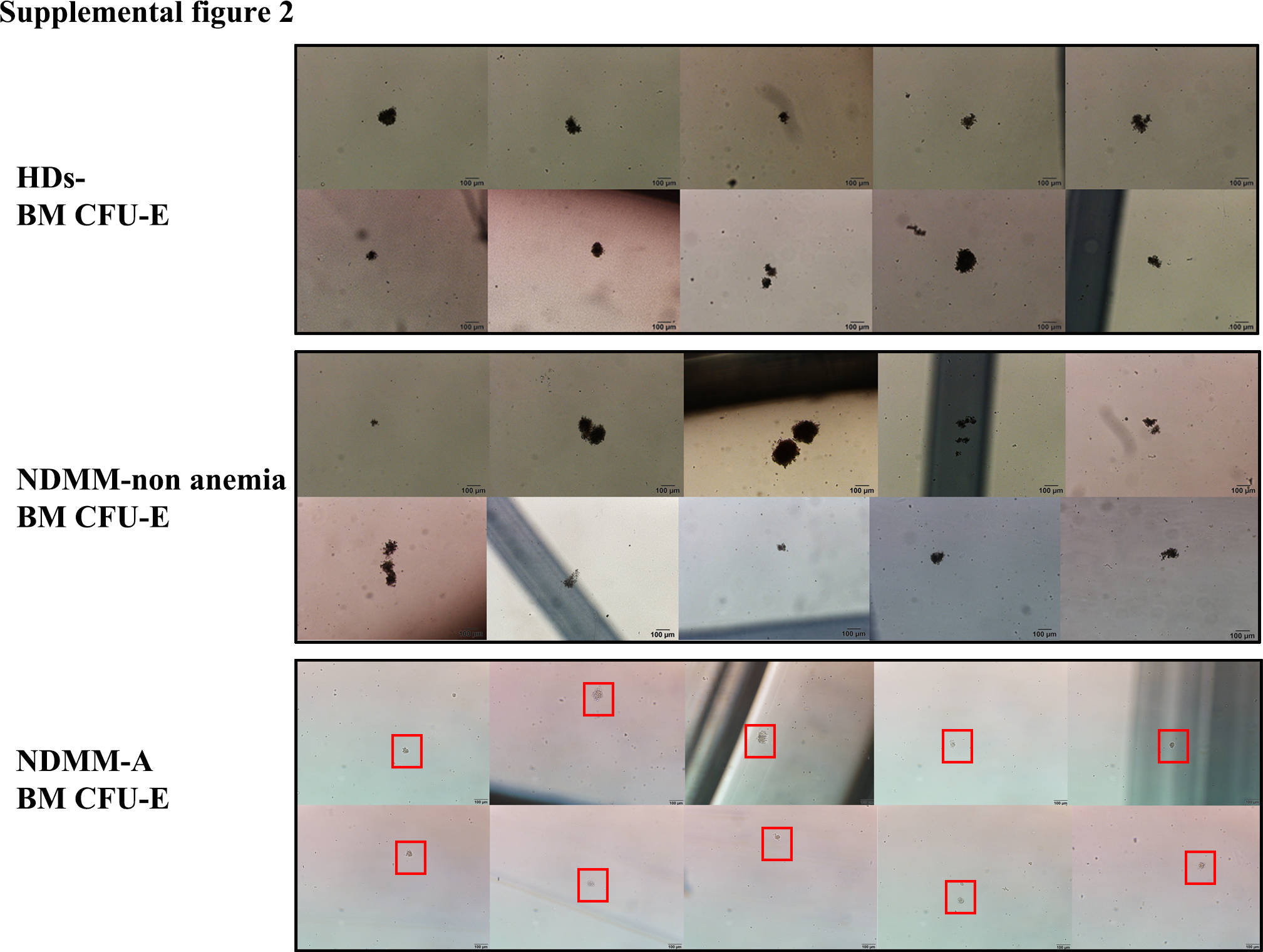

Supplement: Supplementary file 4 [file Image2.tif]

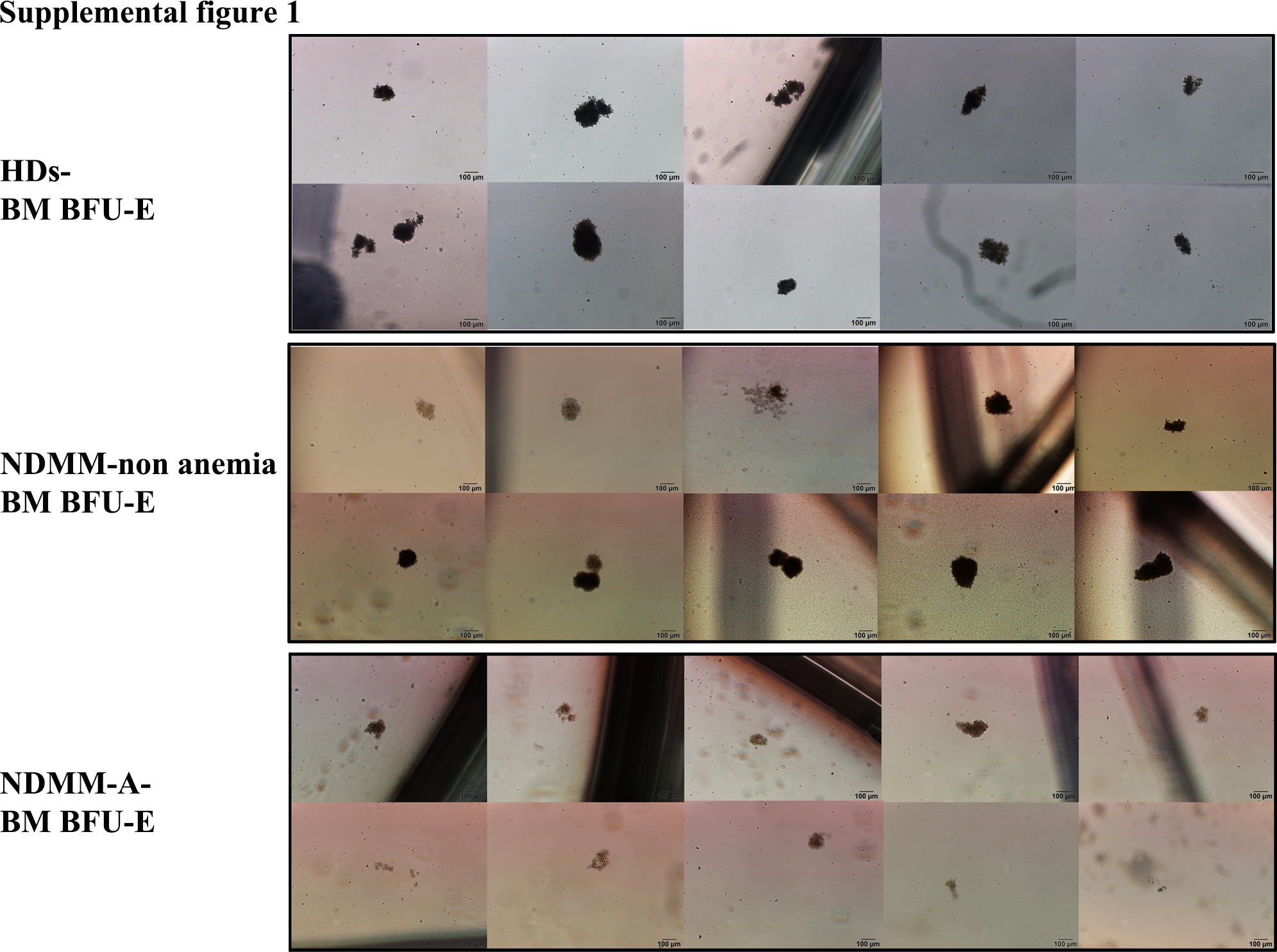

Supplement: Supplementary file 5 [file Image1.tif]

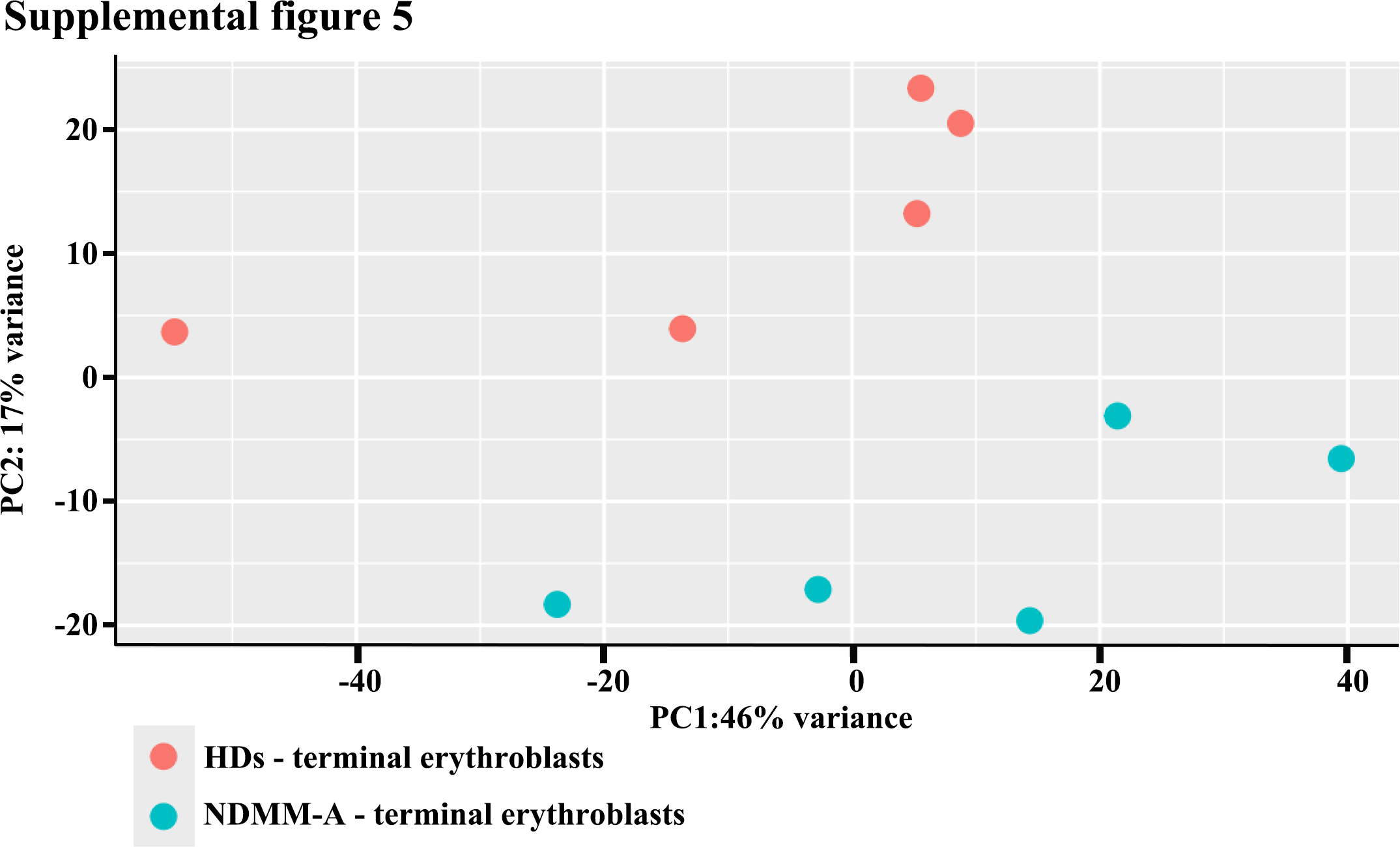

Supplement: Supplementary file 7 [file Image5.tif]
